# Gustatory polymorphism mediates a new adaptive courtship strategy

**DOI:** 10.1098/rspb.2022.2337

**Published:** 2023-03-29

**Authors:** Ayako Wada-Katsumata, Eduardo Hatano, Coby Schal

**Affiliations:** Department of Entomology and Plant Pathology, North Carolina State University, Raleigh, NC 27695, USA

**Keywords:** German cockroach, gustation, aversion, courtship, evolution, nuptial gift

## Abstract

Human-imposed selection can lead to adaptive changes in sensory traits. However, rapid evolution of the sensory system can interfere with other behaviours, and animals must overcome such sensory conflicts. In response to intense selection by insecticide baits that contain glucose, German cockroaches evolved glucose-aversion (GA), which confers behavioural resistance against baits. During courtship the male offers the female a nuptial gift that contains maltose, which expediates copulation. However, the female's saliva rapidly hydrolyses maltose into glucose, which causes GA females to dismount the courting male, thus reducing their mating success. Comparative analysis revealed two adaptive traits in GA males. They produce more maltotriose, which is more resilient to salivary glucosidases, and they initiate copulation faster than wild-type males, before GA females interrupt their nuptial feeding and dismount the male. Recombinant lines of the two strains showed that the two emergent traits of GA males were not genetically associated with the GA trait. Results suggest that the two courtship traits emerged in response to the altered sexual behaviour of GA females and independently of the male's GA trait. Although rapid adaptive evolution generates sexual mismatches that lower fitness, compensatory behavioural evolution can correct these sensory discrepancies.

## 1. Introduction

Under intense natural and anthropogenic selection, adaptive traits can evolve rapidly in the selected populations [1–5]. However, when behavioural traits are abruptly modified by directional selection, other behaviours that affect fitness might be distorted. A compelling example is *Teleogryllus oceanicus* (the Pacific field cricket), in which males evolved a new behaviour under strong selection by the invasive parasitic fly *Ormia ochracea*. The parasitic fly uses its acute hearing to find male crickets that sing to attract females and lays its eggs on the male; the emerging fly larvae then devour the male cricket. In response to this intense selection, the frequency of the *flatwing* mutation, which results in the loss of acoustic sexual signalling, dramatically increased in cricket populations; the flatwing trait is adaptive because males evade parasitoid attacks [6–8]. However, this emergent trait also reduces the male's courtship success, and therefore results in a trade-off between ecological selection and sexual selection [9]. Notably, non-singing males can engage in alternative reproductive strategies, such as intercepting females that approach signalling males.

Yet, it is poorly understood how animals that use other sensory modalities overcome the conflicts of an emergent trait that is concurrently highly adaptive for one critical behaviour and highly maladaptive for another. To address this question, in this study we used the German cockroach, *Blattella germanica*, to investigate how their foraging and courtship are differentially influenced by a recently evolved gustatory polymorphism. Gustatory responses to sugars in the German cockroach drive both foraging and courtship behaviours [10]. Highly appetitive sugars such as glucose, maltose and maltotriose are detected by gustatory receptor neurons (GRNs) [11,12]. Males exploit the females’ fondness of sugars by producing a nuptial secretion during courtship that contains maltose and related oligomers [13–21]. However, as an important worldwide public health pest [22,23], the German cockroach has been exposed to insecticides formulated in glucose-containing baits. Such intense anthropogenic selection favoured changes in the gustatory sense in multiple cockroach populations that evolved glucose-aversion (GA) as a behavioural resistance mechanism that enables them to avoid eating these baits [24,25]. This aversion is mediated by GRNs tuned to detect glucose as a deterrent [12,26,27]. However, GA females experience lower mating success with wild-type (WT) males [28,29]. During courtship, males offer a highly palatable tergal secretion to attract and place the female in the proper position for copulation. The male raises his wings and exposes a nuptial gift in his highly specialized tergal glands; the female mounts the male's dorsum and feeds on the tergal secretion [10]. A critical function of the male's nuptial gift is to arrest the female long enough for the male to extend his abdomen under the female and engage her genitalia. Short nuptial feeding by the female interrupts the genitalia grasping behaviour by the male [29]. Thus, the male accrues reproductive benefits by exploiting the female's gustatory preference and gains competitive advantage in courtship and ultimately reproduction. However, glucosidases in cockroach saliva rapidly hydrolyse oligosaccharides in the nuptial secretion into glucose [29,30]. The courted GA female briefly accepts the male's nuptial gift, but then detects glucose and rejects it, dismounts the male, and interrupts courtship before the male can grasp the female genitalia; WT females, on the other hand, engage in longer nuptial feeding and experience greater copulation success [29].

Remarkably, recent studies [28,29] demonstrated that GA males experienced greater success in copulation with GA females than WT males. In this study, we hypothesized that a variant adaptive courtship strategy evolved in GA males during the approximately 150 generations of selection with glucose-containing toxic bait in the laboratory. If so, we also hypothesized that this emergent male sexual behaviour might be genetically linked to the GA trait. Comparative analysis of courtship behaviour revealed that GA males express more rapid courtship behaviour than WT males. Analysis of the sugar composition of their nuptial secretion showed that GA males adjusted the sugar blend of their nuptial secretion to restore their mating success with GA females. Recombinant lines of WT and GA cockroaches showed no evidence of genetic linkage between the variant courtship traits and the GA trait. The results suggest that the GA males’ courtship traits developed under sexual selection imposed by the new gustatory trait of GA females. Although rapid evolution of a chemosensory trait that is adaptive for foraging may be maladaptive in courtship [29], the compensatory evolution of behaviour and a modified nuptial gift mitigated the mismatch between male signals and female preference and restored reproductive fitness.

## 2. Material and methods

### (a) Cockroach strains

All cockroaches were maintained on rodent diet (Purina 5001, PMI Nutrition International, St Louis, MO, USA) and insect rearing and all experiments were carried out at 27°C, approximately 40% relative humidity and a 12 : 12 h L : D cycle. The WT strain (Orlando Normal, collected in Florida in 1947) is a standard strain used in many investigations involving the German cockroach. The GA strain (T-164, obtained in Florida in 1989) was selected in the laboratory with glucose-containing toxic baits every generation or every few generations to fix the homozygous GA trait in the population (approximately 150 generations as of 2020). As described in detail in our previous study [29] and in the electronic supplementary material, recombinant colonies were initiated in 2013 by crossing 10 pairs of WT males × GA females and 10 pairs of GA males × WT females to homogenize the genetic backgrounds of the two strains. At the F_8_ generation (free bulk mating without selection), 400 cockroaches were separated into two groups: glucose-accepting and glucose-rejecting. These groups were bred for three more generations with rodent diet, which is typically used for maintaining the cockroach strains. The glucose-rejecting group received artificial selection with a glucose-containing toxic bait in the first and fifth instar stages at each generation. Then, 200 cockroaches from each group were assayed in the F_11_ generation and backcrossed to confirm the homozygous glucose-accepting (aa) and glucose-averse (AA) lines. Similar results were obtained in both directions of the cross, confirming previous findings of no sex linkage of the GA trait [25]. GA homozygosity of these two lines was confirmed by a *backcross assay* to obtain WT_aa (homozygotes, glucose-accepting) and GA_AA (homozygotes, glucose-averse). We cultured these two lines for three more generations (F_14_) and used them for this study. To determine the effective concentration (EC_50_ values) for glucose acceptance and glucose rejection in females, F_14_ WT_aa and F_14_ GA_aa were tested in the *acceptance–rejection assay* (see below).

### (b) Mating bioassay

To extract differences in courtship behaviours between WT and GA males, a male and female pair was placed in a Petri dish with fresh water and a piece of rodent food. All pairs were video-recorded with an infrared-sensitive camera (Polestar II EQ610, EverFocus Electronics) coupled to a data acquisition board and analysed with frame-by-frame capable software (NV3000, AverMedia Information). Recordings were classified into two groups: successful mating and failed mating. Distinct behavioural events included contact, wing raising, nuptial feeding and copulation [29], and they were analysed using four parameters: latency of wing raising display in males (s), nuptial feeding duration in females (s), copulation latency in males (s) and copulation duration (min). A successful mating sequence was defined as the courtship events from contact to copulation in the mated group.

We conducted two types of mating bioassays. In a standard mating bioassay to compare the courtship performance of WT and GA males, four types of pairs were observed using sexually mature males (10–12 days old) and sexually mature females (5–7 days old) : WT males × WT females (*n* = 52 pairs); GA males × GA females (*n* = 80 pairs); GA males × WT females (*n* = 52 pairs); and WT males × GA females (*n* = 67 pairs). To evaluate the association of male courtship traits and the GA trait, the courtship performance of males from the F_14_ recombinant lines (WT_aa males and GA_AA males) was tested. Before starting the mating bioassays using the recombinant lines, 7-day-old males from each line were subjected to the following *two-choice feeding assay***:** 10 males (WT_aa males, non-starved; GA_AA males, 1 day starved without water) were placed in a Petri dish (90 mm dia.× 15 mm) containing two agar discs. One disc contained 1% agar and 1 mmol l^−1^ of food-grade Allura Red AC, and the second disc contained 1% agar, 0.5 mmol l^−1^ of food-grade Erioglaucine disodium salt and 1000 mmol l^−1^ glucose. The assay duration was 2 h during the dark phase of the insects’ L : D cycle. After each assay, the colour of the food dye consumed by individuals was visually inspected by observing the abdomen under a microscope to assess their glucose-appetitive or glucose-aversive behavioural traits. Then, individual males were paired with GA females (*n* = 25 pairs using WT_aa males, 24 pairs using GA_AA males). To present sexually mature 5-day-old females to the males when they were 12 days old, 0-day-old females were paired with 7-day-old males, and we video-recorded them until the females became 9 days old.

### (c) Acceptance–rejection assay

This rapid qualitative assay assessed the instantaneous initial responses (yes-no) of the insects to tastants [26]. Acceptance indicates that the cockroach started drinking. Rejection indicates that the cockroach never initiated drinking. To conduct dose–response studies with phagostimulants and deterrents, adults were deprived of food for 24 h, but supplied with water. When testing deterrents, the cockroaches were deprived of both food and water for 24 h to increase their thirst. The mouthparts were carefully touched with a drop of stimulus solution coloured with 1 mmol l^−1^ blue food dye (erioglaucine) in a sequence from the lowest to the highest concentration. The percentage of positive responders was defined as the number of insects accepting a tastant/total number of insects tested. The EC_50_ for each tastant was obtained from dose–response curves using this assay. To obtain EC_50_ estimates for glucose in the recombinant lines, 4-day-old females (30 F_14_ WT_aa were non-starved and 30 F_14_ GA_aa were starved for 1 day without water) were tested with a concentration series of glucose (0, 10, 30, 100, 300 and 1000 mmol l^−1^). The EC_50_ values of WT females and GA females were obtained from previous work [29]. To estimate the EC_50_ of female acceptance of nuptial secretions from WT males and GA males, each nuptial secretion was diluted with high performace-liquid chromatography (HPLC)-grade water (Fisher Scientific) to 0.001, 0.01, 0.03, 0.1, 0.3 and 1 male-equivalents µl^−1^, and 20 non-starved 4-day-old WT females and GA females were tested.

### (d) Consumption assay

To compare the amount of nuptial secretion consumed by non-starved 5-day-old WT females and GA females, nuptial secretions from WT males and GA males were diluted, respectively, with HPLC-grade water to 0.1 male-equivalents µl^−1^. We used the same methodology as in the acceptance–rejection assay, but continued to observe individual females until they stopped drinking, which we considered a single bout of feeding (*n* = 10 WT females and 10 GA females).

### (e) Effect of saliva on female acceptance of nuptial secretion

Previous studies showed that the saliva of both WT females and GA females hydrolyses the nuptial oligosaccharides presented by WT males, causing the release of glucose, which deters GA females from feeding on the male's offerings [29,30]. In this study, to determine if exposure of WT male and GA male nuptial secretions to female saliva results in differential release of glucose, we mixed either WT male or GA male nuptial secretions with either WT female or GA female saliva. Additionally, we tested the feeding responses of WT females and GA females to WT male or GA male nuptial secretions mixed with female saliva.

The nuptial secretion was collected from males by the following method: five 10–12 days-old males were placed in a container (95 × 95 × 80 mm) with a 5-day-old GA female. After the males displayed wing-raising courtship behaviour towards the female, individual males were immediately decapitated and the nuptial secretion in their tergal gland reservoirs was drawn into a calibrated borosilicate glass capillary (76 × 1.5 mm) under a microscope. The nuptial secretions from 30 males were pooled in a capillary and stored at –20°C until use.

Saliva from 5-day-old WT females and GA females was collected by briefly anaesthetizing individual females with carbon dioxide under the microscope and the side of the thorax was gently squeezed. A droplet of saliva that accumulated on the mouthparts was then collected into a microcapillary (10 µl, Kimble Glass). Fresh saliva was immediately used in experiments.

For quantification of saliva-catalysed hydrolysis of WT male and GA male tergal secretion, the samples were prepared as follows: 1 µl of GA female saliva was mixed with 1 µl of 10 male-equivalents µl^−1^ HPLC-grade water. We incubated the mixtures for 0, 5, 10 and 300 s at 25°C, and added 4 µl of methanol to stop enzyme activity (*n* = 5 for each incubation time). Each sample contained the nuptial secretions of five males to optimize the quantification of sugars. For statistical analysis, the amounts of various sugars were divided by five to obtain the amounts in one male (1 male-equivalent).

We used the acceptance–rejection assay as follows: 1 µl of either saliva from WT females or GA females or HPLC-grade water was added to nuptial secretion (1 µl representing 10 male-equivalents). Then, 8 µl of HPLC-grade water was added to the mix, yielding a final concentration of the test solution of 1 male-equivalent µl^−1^ of nuptial secretion in a total volume of 10 µl. The mix of saliva and nuptial secretion was incubated for 300 s at 25°C, then the acceptance–rejection assay was carried out with 5-day-old WT females and GA females (*n* = 20–33 each). Saliva alone does not affect acceptance or rejection of stimuli [29]. Additionally, to confirm the contribution of salivary glucosidases in salivary digestion of nuptial secretion, we added the glucosidase inhibitor acarbose to prevent the hydrolysis of sugars and tested the incubation products in the acceptance–rejection assay. Test solutions were prepared as follows: 1 µl of either saliva from 5-day-old GA females or HPLC-grade water was mixed with 0.5 µl of either 250 µmol l^−1^ of acarbose or HPLC-grade water. This mixture was added to 0.5 µl of 10 male-equivalents of nuptial secretion. HPLC-grade water was added for a total volume of 10 µl and a final concentration of 1 male-equivalent µl^−1^. As above, the mixture was incubated for 300 s at 25°C and offered to 5-day-old GA females (*n* = 20) in acceptance–rejection assays. At the concentrations we used, acarbose does not affect acceptance or rejection of tastants [29].

### (f) Sugar analysis using gas chromatography-mass spectrometry

We focused the gas chromatography-mass specrometry (GC-MS) analysis of nuptial secretion on glucose, maltose and maltotriose of WT males, GA males, WT_aa males and GA_AA males. Details of the methods are described elsewhere [29], but additional details are provided in the electronic supplementary material. d-(+)-maltose (Fisher Scientific), d-(+)-glucose and maltotriose (Sigma-Aldrich) were used as calibration standards and sorbitol was used as an internal standard for each sample.

*N*-methyl-*N*-(trimethylsilyl)trifluoroacetamide (MSTFA; Sigma-Aldrich) was used for derivatization of sugars, and samples were analysed by GC-MS, as detailed in [29] and in the electronic supplementary material.

For the quantification of saliva-catalysed hydrolysis of WT male and GA male tergal secretion, 1 µl of GA female saliva was mixed with 1 µl of 10 male-equivalents µl^−1^ HPLC-grade water. We incubated the mixtures for 0, 5, 10 and 300 s at 25°C, and added 4 µl of methanol to stop enzyme activity (*n* = 5 each incubation time). Each sample contained the nuptial secretions of five males to optimize the quantification of sugars. For statistical analysis, the amounts of various sugars were divided by five to obtain the amounts in one male (1 male-equivalent).

### (g) Statistical analysis

The number of replicates and sample size are shown in the section describing the experimental details. In summary, the samples sizes were: mating bioassays, *n* = 24–80; feeding assays using females, *n* = 20–30; sugar analysis, *n* = 5–7. All statistical analyses were conducted in Prism (GraphPad Software, San Diego, CA, USA). Results of bioassays and sugar analyses are indicated in figures by the means, standard errors and all data points. We used the *χ*^2^-test with Holm's method for post hoc comparisons, *t*-test, and ANOVA followed by Tukey's HSD test (all *α* = 0.05), as noted in each section describing the experimental details, results, and in the electronic supplementary material, tables S1–S9.

## 3. Results

### (a) Short copulation latency in glucose-aversion males improves mating success with glucose-aversion females

**Figure 1.**
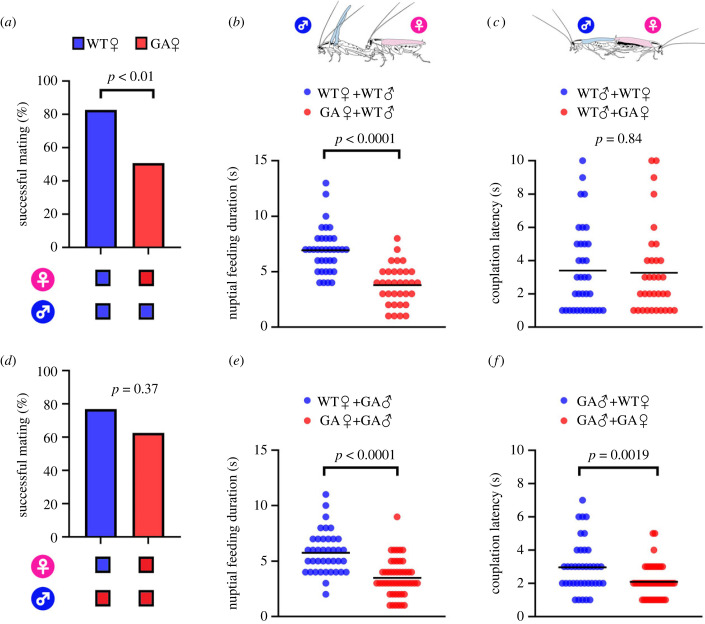
Glucose-averse German cockroach males (♂) improve their mating success with short copulation latency. Successful mating in no-choice mating assays of (*a*) WT♂ (*n* = 52 for WT females (♀), 67 for GA♀) or (*d*) GA♂ (*n* = 52 for WT♀, 80 for GA♀) (*χ*^2^-test). Nuptial feeding duration of (*b*) WT♀ and GA♀ for WT♂ (*n* = 36 in WT♀, 33 in GA♀) or (*e*) WT♀ and GA♀ for GA♂ (*n* = 39 in WT♀, 49 in GA♀) courting either WT♀ or GA♀ (*t*-test). Copulation latency of (*c*) WT♂ (*n* = 35 with WT♀, 33 with GA♀) or (*f*) GA♂ (*n* = 38 with WT♀, 48 with GA♀) (*t*-test). *p*-values are indicated in each graph.

Successful mating in the German cockroach almost invariably requires that the female mount the male's dorsum and feed on the male's tergal secretion, which enables the male to extend his abdomen and grasp the female's genitalia. As previously shown [29], short nuptial feeding can result in failure to mate because males do not have enough time to grasp the female's genitalia. In reciprocal no-choice mating assays, mating success of WT males paired with GA females was significantly lower than with WT females (*χ*^2^-test, *χ*_2_^2^ = 11.7, *p* < 0.01) (figure 1*a*; electronic supplementary material, table S1). When paired with WT males, nuptial feeding by GA females was significantly shorter than by WT females (*t*-test, *t*_67_ = 6.8, *p* < 0.0001) (figure 1*b*). However, no difference was found in copulation latency of WT males paired with either WT females or GA females (figure 1*c*). By contrast, GA males had similar mating success with WT females and GA females (figure 1*d*). Yet, when paired with GA males, nuptial feeding by GA females was significantly shorter than by WT females (*t*-test, *t*_86_ = 6.0, *p* < 0.0001) (figure 1*e*). GA males paired with GA females had significantly shorter copulation latency than with WT females (*t*-test, *t*_84_ = 3.2, *p* < 0.01) (figure 1*f*). These results indicate that GA females engage in short nuptial feeding with both WT males and GA males. However, whereas GA males shorten their copulation latency with GA females, WT males fail to do so and often fail to mate with GA females. Variation in two other parameters of male sexual behaviour—latency of the wing-raising display and copulation duration—did not contribute to success in securing a copulation by GA males (electronic supplementary material, table S1).

### (b) The tergal secretion of glucose-aversion males contains more palatable sugar components

**Figure 2.**
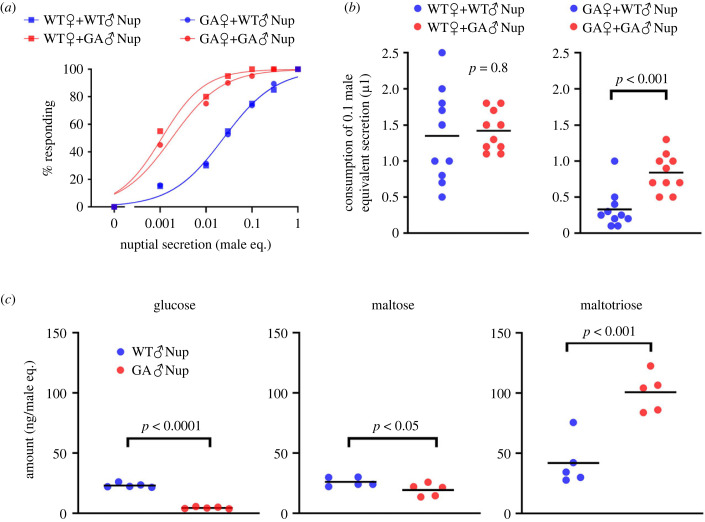
Glucose-averse *B. germanica* males (♂) produce a more attractive nuptial secretion than WT♂. (*a*) Dose–response (feeding acceptance) curves for WT females (♀) and GA♀ responding to WT_aa♂ nuptial secretion (WT♂Nup) or GA_AA♂ secretion (GA♂Nup) (*n* = 20 for each). None of the females responded to water (0 male-equivalent), the vehicle used for nuptial secretion. (*b*) Consumption by WT♀ and GA♀ of WT♂Nup and GA♂Nup in a single feeding bout (*n* = 10 for each) (*t*-test). GA♀ consumed significantly less WT♂Nup than GA♂Nup. (*c*) Sugar contents of WT♂Nup and GA♂Nup (*n* = 5 for each) (*t*-test). *p*-values are indicated in each graph. WT♂Nup contained significantly more glucose and maltose, whereas GA♂Nup contained significantly more maltotriose.

To determine if females respond to WT male and GA male nuptial secretions differently, we performed dose–response acceptance–rejection assays, which revealed the initial instantaneous response of females to tastants. The nuptial secretion of GA males stimulated feeding in both WT females and GA females at significantly lower concentrations than the nuptial secretion of WT males (extra sum-of-squares *F*-test, *F*_3,20_ = 57.9, *p* < 0.0001) (figure 2*a*; electronic supplementary material, table S2). In the consumption assays, which measured the total amount consumed in a single feeding bout, WT females equally consumed the nuptial secretions of both WT males and GA males. By contrast, GA females ingested significantly less nuptial secretion from WT males than GA males; GA females stopped consuming the WT male nuptial secretion earlier than the GA male nuptial secretion (*t*-test, *t*_18_ = 4.3, *p <* 0.001) (figure 2*b*; electronic supplementary material, table S2). These results indicate a mismatch between the taste preferences of GA females and the composition of the WT male secretion, whereas the quality of the secretion of GA males resulted in sustained feeding by GA females.

Sugar analysis using GC-MS revealed significant differences in the tergal secretions of WT males and GA males (figure 2*c*; electronic supplementary material, table S3). We focused on glucose, maltose and maltotriose, representing increasing complexity of the sugar; previous work did not report the existence of glucose in the nuptial secretion, but maltose and maltotriose were mentioned as important tergal secretion components and phagostimulants [16–21]. WT male tergal secretion contained 5-fold more glucose and 1.3-fold more maltose than the secretion of GA males (*t*-test, *t*_8_ = 21.0, *p* < 0.0001 for glucose; *t*_8_ = 2.3, *p* = 0.049 for maltose). Conversely, GA males contained 2.5-fold more maltotriose than WT males (*t*-test, *t*_8_ = 5.2, *p* < 0.001).

### (c) Impact of salivary digestion on nuptial feeding

**Figure 3.**
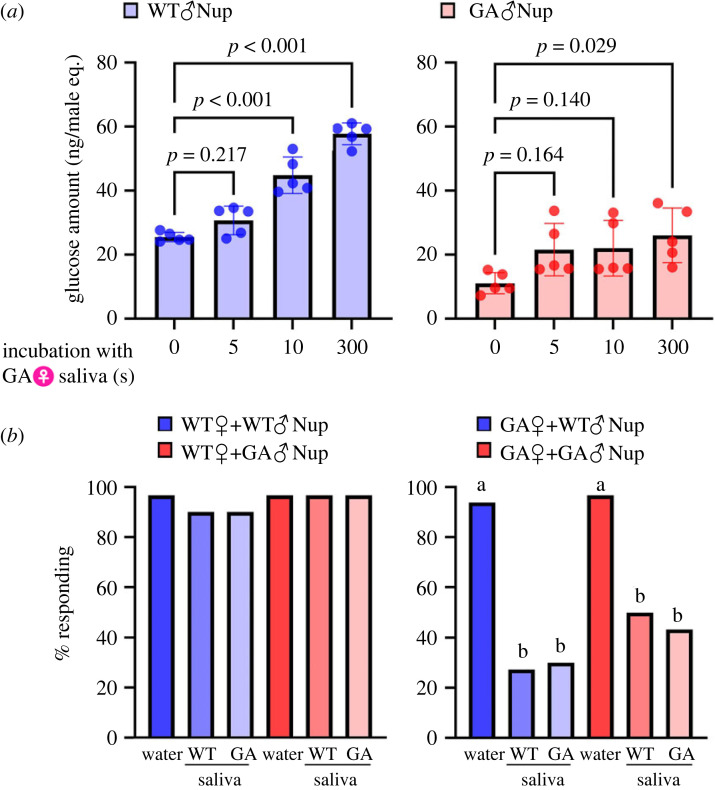
Salivary digestion releases less glucose from the nuptial secretion of glucose-averse *B. germanica* males (♂) than of WT♂. (*a*) Time-course of glucose accumulation when 1 male-equivalent of WT_aa♂ secretion (WT♂Nup) or GA_AA♂ secretion (GA♂Nup) was incubated with the saliva of GA females (♀) (*n* = 5 for each) (one-way ANOVA, *p*-values are indicated in each graph). Addition of saliva increased the glucose concentration in both WT♂Nup and GA♂Nup, but less glucose was released in GA♂Nup. (*b*) Saliva of WT♀ and GA♀, mixed with WT♂Nup or GA♂Nup, convert phagostimulants to deterrents that interrupt nuptial feeding of GA♀ but not WT♀ (*n* > 20 for each) (*χ*^2^-test, different letters indicate significant differences).

Previous studies showed that the saliva of both WT females and GA females hydrolyses WT male nuptial oligosaccharides, causing the release of glucose, which deters GA females from feeding on the male's offerings [29,30]. GC-MS analysis revealed that exposure to saliva released glucose from the nuptial secretions of both WT males and GA males (figure 3*a*; electronic supplementary material, table S4). However, significant amounts of glucose were released from the secretion of GA males only after 300 s of incubation with saliva, whereas the secretion of WT males released significant amounts of glucose between 5 and 10 s of incubation with saliva (one-way ANOVA, WT nuptial secretion, *F*_3,16_ = 64.2, *p* < 0.001; GA nuptial secretion, *F*_3,16_ = 3.6, *p* = 0.037).

To confirm that the saliva disrupts nuptial feeding of GA females, we incubated nuptial secretion with saliva for 300 s, and assessed its acceptability with the acceptance–rejection assay (figure 3*b*; electronic supplementary material, table S5). WT females accepted the nuptial secretions of both WT males and GA males, either with or without incubation with saliva. However, GA females accepted the intact secretions more than the incubated secretion (*χ*^2^test, *χ*^2^_5_ = 61.3, *p* < 0.01). Additionally, when acarbose was co-incubated with saliva for 300 s, more GA females accepted the incubated secretion. These results confirmed that salivary alpha-glucosidases effectively reduced the palatability of tergal secretions to GA females, resulting in reduced acceptance by GA females of both WT male and GA male secretions (*χ*^2^-test, WT nuptial secretion, *χ*^2^_3_ = 42.5, *p* < 0.0001; GA nuptial secretion, *χ*^2^_3_ = 30.4, *p* < 0.0001) (electronic supplementary material, table S6). However, the secretion of GA males is more resilient than the secretion of WT males to hydrolysis by the saliva of GA females. Considering that maltotriose is more resilient than maltose to hydrolysis by the saliva of GA females [29] and that the nuptial secretion of GA males elicits greater acceptance by the female (figure 2*a,b*), high maltotriose in GA males secretion appears to be highly adaptive for prolonging GA female nuptial feeding.

### (d) No linkage of the glucose-aversion trait with the two variant courtship traits of glucose-aversion males

**Figure 4.**
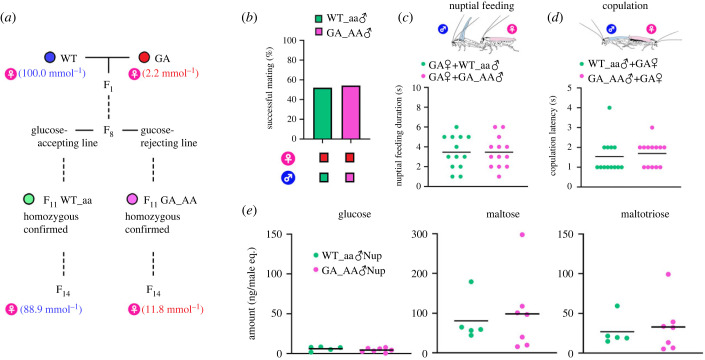
Two emergent male (♂) courtship traits are not genetically linked with the GA trait in *B. germanica*. (*a*) The experimental design used to generate four recombinant lines. The EC_50_ values for glucose acceptance in WT female (♀) and WT_aa♀, and glucose rejection in GA♀ and GA_AA♀ are shown in parentheses. (*b*) Successful mating in no-choice mating assays of WT_aa♂ and GA_AA♂ and GA♀ (*n* > 24) (*χ*^2^-test). (*c*) Nuptial feeding duration of GA♀ with either WT_aa♂ or GA_AA♂ (*n* = 13 for each) (*t*-test). (*d*) Copulation latency of WT_aa♂ and GA_AA♂ with GA♀ (*n* = 13 for each) (*t*-test, *α* = 0.05). (*e*) Sugar contents of WT_aa♂ secretion (WT_aa♂Nup) and GA_AA♂ secretion (GA_AA♂Nup) (*n* > 5 for each) (*t*-test). *p*-values are not shown because there were no significant differences in each graph.

To test for possible pleiotropy between the GA trait and the two emergent male courtship traits, namely lower copulation latency and altered ratio of three sugar components in GA males, we generated a recombinant line of WT (obtained in 1947) and GA (obtained in 1989) cockroaches. After eight generations of admixture, we created two genotypes by screening their feeding responses to glucose (figure 4*a*). The WT_aa line was glucose-accepting and the GA_aa line was selected with a glucose-containing toxic bait until F_11_. These divergent recombinant lines were in culture three more generations (F_14_) and we tested their feeding responses to glucose (electronic supplementary material, table S7). We hypothesized that if the two courtship traits of GA males are genetically linked with the GA trait, this linkage should be apparent despite the short history of selection (F_8_–F_14_). This hypothesis predicts that the variant courtship traits will be evident only in GA_AA males. Conversely, independence of the male courtship traits and the GA trait would suggest that the two male traits emerged in response to selection pressure imposed by the altered gustatory preferences of GA females, and independently of the male's GA trait. The latter hypothesis predicts an evolutionary response of GA males to selection pressure imposed by GA females as they replace WT females under directional selection by glucose-containing toxic baits. In this case, similar courtship traits should be evident in both WT_aa males and GA_AA males.

Turning to the interactions of WT_aa males and GA_AA males with GA females, we found no difference between the two lines in their respective mating success, duration of nuptial feeding by GA females, and copulation latency (figure 4*b–d*; electronic supplementary material, table S8). This finding indicates that short copulation latency as a courtship behavioural trait is not genetically linked with the GA trait. Sugar analysis revealed no significant differences in the secretions of WT_aa males and GA_AA males (figure 4*e*; electronic supplementary material, table S9). These results indicate that the production of these three sugars in the tergal glands of the German cockroach is regulated independently of the GA trait.

## 4. Discussion

Rapid and strong ecological selection pressure can lead to new physiological and behavioural phenotypes that may conflict in their adaptive values in different physiological and behavioural contexts [31]. We previously demonstrated that a highly adaptive polymorphism in the gustatory modality evolved under strong selection pressure of glucose-containing toxic baits; however, it created an intersexual conflict in German cockroach courtship [29]. During courtship, the male cockroach offers the female a sugar-containing nuptial gift. However, female saliva releases glucose from more complex oligosaccharides, transforming the nuptial offering into an aversive stimulus that causes GA females to reject courting males. In a previous study [29], we demonstrated that GA females abate their lower mating success by reducing the alpha-glucosidase activity of their saliva, so less of the tasty maltose and matotriose in the male's nuptial gift is hydrolysed to aversive glucose. Here, we demonstrated that GA males also express two emergent courtship-related traits that mitigate their lower mating success. The tergal secretions of GA males contain less maltose and more maltotriose than in WT males. Considering that both WT and GA females have higher feeding sensitivity to maltotriose than maltose and other sugars [11,12,26,29,30], these results indicate that the sugar blend of GA males is more palatable to all females than the sugar blend of WT males. Moreover, because maltotriose is more recalcitrant to hydrolysis by salivary enzymes than maltose, it also retains its phagostimulatory properties long enough to allow the male to engage the female's genitalia. GA males also evolved a more rapid copulatory response, so they reach out to and engage the female genitalia before the GA female interrupts her nuptial feeding.

Gifting of significant nutrient sources from males to females has been reported in many insect species, including predatory dance flies [32] and bushcrickets [33], where substantial nutritional gifts may be spermatophores, prey, various nuptial secretions, humoral gifts of body parts or blood, and even the female feasting on the male during or after copulation. In other species, gifts might provide context-dependent benefits to females. In *Drosophila subobscura*, for example, healthy males in good dietary condition produce more regurgitated gifts during courtship than do starved or nutrient-deficient males [34]. The female's nutritional condition also affects courtship outcomes. Male regurgitant gifts have no effect on the fecundity of females in good condition, but fecundity of females in poor condition increases to the level of females in good condition by feeding on regurgitated gifts from males. Thus, it appears that the male exploits the female's gustatory preference by offering her a courtship gift that carries some value only when the female is in a poor nutritional state [34].

In some insects, the nuptial offerings may represent minimal benefits or even valueless tokens (e.g. rocks), yet somehow they manipulate the female to accept the courting male [35]. The German cockroach nuptial gift appears to represent this syndrome, as the quantity of the tergal secretion is minimal, the architecture of the reservoirs makes it difficult for the female to obtain the secretion, and females in good or poor nutritional condition do not appear to accrue fitness benefits from feeding on the male's secretion [36]. Therefore, in the German cockroach, the male's gifting strategy appears to exploit sensory biases in females. Although both sexes cooperate in courtship and ultimately in mating, male exploitation of the female's gustatory preferences may lead to sexual conflict, as directional selection causes female gustatory preferences and male nuptial secretions to diverge. Variant courtship traits are expected to emerge in both males and females to ameliorate these mismatches and maximize mating success.

### (a) Emergent courtship behaviours mitigate the adverse effects of the glucose-aversion trait

Conflicts and trade-offs between new adaptive traits and WT traits are prominent in anthropogenic selection, such as the emergence insecticide resistance that may compromise size, locomotor activity, fecundity or longevity [37]. For example, the DDT resistance allele of *Drosophila melanogaster* is associated with phenotypes that have smaller body size and lower aggressive performance [38]. Also in *D. melanogaster*, a single amino acid residue substitution in the acetylcholine receptor increased resistance to neonicotinoid insecticides, but was accompanied by a significant reduction in fitness [39]. These and other studies, like ours [29], document that emergent adaptive traits can cause lower mating success, especially under strong intrasexual reproductive competition.

In the case of the German cockroach, there were no differences in mortality, development and fecundity between WT and GA cockroaches in a glucose-free foraging environment [29]. However, when glucose is a prominent food source in the environment, GA cockroaches experience significant deficits in growth, development and reproduction. These costs are mitigated under anthropogenic selection with glucose-containing toxic baits, as WT cockroaches die and GA cockroaches avoid eating the bait. Nevertheless, this emergent adaptive trait is severely debilitating in sexual interactions, especially between GA females with WT males. Thus, the GA adaptive gustatory polymorphism is shaped by both ecological and sexual selection.

In this study, we addressed the latter—has sexual selection pressure compelled GA males to express alternative courtship strategies to overcome the sensory conflict between efficient foraging and mating success? Since our previous studies showed that GA males were more successful than WT males in mating with GA females [28,29], we hypothesized that in a homozygous GA population, GA males might have evolved alternative courtship strategies that improve their mating success with GA females.

Analysis of courtship sequences revealed two variant courtship traits in GA males that we did not observe in WT males: short copulation latency and maltotriose-rich nuptial secretion. Unlike WT males that required 3.3–3.9 s of female nuptial feeding to lock genitalia with the female and initiate copulation (figure 1*c*), GA males grasped the GA female's genitalia more rapidly (within 2.2 s), before the taste of glucose caused the female to dismount the male's dorsum (figure 1*f*). Moreover, GA males produce not only less glucose, consistent with its deterrence to GA females, but they also enrich their nuptial secretion with more maltotriose (figure 2*c*). Maltotriose is highly phagostimulatory, eliciting feeding responses in sexually mature WT and GA females at very low concentration (EC_50_ = 1.6–2.3 mmol l^−1^) [30]. Importantly, maltotriose is also more recalcitrant than maltose to hydrolysis by salivary enzymes [29], so it retains its phagostimulatory properties longer during nuptial feeding. Overall, the modified nuptial secretion of GA males is a better match than the secretion of WT males with the new taste preferences of GA females.

Interestingly, experiments using recombinant lines suggested no linkage between these two GA male-specific traits and the GA trait, which is expressed by both sexes and all life stages of the German cockroach. We suggest that the emergent GA male behaviour and the production of a modified nuptial secretion evolved independently in response to sexual selection by the transformed gustatory preferences of GA females. The recombinant lines will be essential for future investigations of the genetic mechanisms that underlie these changes in GA males. We suspect that selection for only six generations (F_8_–F_14_) to generate the WT_aa and GA_AA males was not sufficient for the alternative traits to appear. Moreover, it is important to note that our selection was based on gustatory responses to glucose, and not on copulation latency or the chemistry of the nuptial secretion.

### (b) Assortative mating-facilitated introgression of the glucose-aversion trait into cockroach populations

Although the origin of the GA trait is unclear, to develop ecologically sound pest management strategies it is imperative to understand how the GA trait introgresses into and is maintained in various field populations of the German cockroach. An important finding of this study is that the GA trait may be introduced into and spread throughout WT populations through sexually biased gene flow. In populations containing a mix of GA and WT cockroaches, WT males experience less mating success with GA females because the females reject glucose in the nuptial secretion and dismount the courting male prematurely. Conversely, GA males successfully mate with both WT and GA females, thus introgressing the GA allele into WT populations. Under strong selection pressure from glucose-containing toxic baits, the WT and heterozygous GA cockroaches (lower sensitivity to glucose as an aversive tastant and thus less rejection of glucose) would be eradicated by the baits, and rapidly be replaced by homozygous GA cockroaches that avoid eating the baits (‘natural' selection). As selection pressure persists, GA females would prefer GA males that express the new adaptive courtship traits (positive-assortative mating under sexual selection), biasing male–male intrasexual contests in favour of GA males, and ultimately driving the population to homozygosity of both GA and the alternative male courtship traits. These genotypes would then be favoured because they restore both foraging and mating success and overall fitness to both sexes.

Sex-biased dispersal of genes is influenced by various factors including the mating system, sex ratio, costs of dispersal, local competition for mates and resources, inbreeding avoidance, habitat persistence and dispersal timing [40–42]. Most theoretical studies of vertebrates agree that in polygynous or promiscuous species, males are predicted to be the more dispersive sex [43]. However, little is known about the patterns of sex-biased dispersal in invertebrates including pest insects carrying adaptive resistance alleles [44,45]. Additionally, most studies have focused on the migration ability of either males or females as a critical factor in gene flow, but the impacts of mating preferences on migration and gene flow are poorly understood. The German cockroach represents a polygynous mating system. However, females are gravid with an egg case for most of their adult lives during which they are sexually unreceptive and more sedentary than males. The male-biased operational sex ratio may lead to male-biased migration of both WT and GA individuals. This male-biased gene dispersal system may enhance the transmission of the GA trait within and between populations, potentially causing the replacement of WT genotypes by GA genotypes. Concurrently, gene flow between WT males and emergent GA populations may be thwarted by assortative mating of GA females. This hypothesis needs to be tested with GA trait phenotyping, gene flow simulations and the use of molecular markers in field studies.

In summary, our previous studies demonstrated the emergence of an adaptive gustatory trait, GA, under ecological selection [12] and discovered that it creates sensory mismatches in intersexual communication [29]. The GA trait represents a gustatory polymorphism that operates adaptively or maladaptively in foraging and reproduction, depending on the selection pressures imposed in different environmental contexts. In this study, we suggest that the highly adaptive value of GA in the anthropogenic environment has selected for alternative male reproductive strategies that mitigate its fitness disadvantages during courtship.

## Data Availability

Data is available from the Dryad Digital Repository: https://doi.org/10.5061/dryad.xpnvx0kk6 [46]. The data are provided in the electronic supplementary material [47].
